# Glycemia Is Related to Impaired Cerebrovascular Autoregulation after Severe Pediatric Traumatic Brain Injury: A Retrospective Observational Study

**DOI:** 10.3389/fped.2017.00205

**Published:** 2017-09-25

**Authors:** Adam M. H. Young, Hadie Adams, Joseph Donnelly, Mathew R. Guilfoyle, Helen Fernandes, Mathew R. Garnett, Marek Czosnyka, Peter Smielewski, Mark Plummer, Shruti Agrawal, Peter J. Hutchinson

**Affiliations:** ^1^Division of Academic Neurosurgery, Department of Clinical Neurosciences, Addenbrooke’s Hospital, University of Cambridge, Cambridge, United Kingdom; ^2^Neurosciences Critical Care Unit, Addenbrooke’s Hospital, University of Cambridge, Cambridge, United Kingdom; ^3^Department of Paediatric Intensive Care, Addenbrooke’s Hospital, University of Cambridge, Cambridge, United Kingdom

**Keywords:** brain, injury, acute, glucose, lactate

## Abstract

**Introduction:**

A strong association exists between hyperglycemia and outcome in pediatric traumatic brain injury (TBI). Herein, we describe observations of serum markers of glucose metabolism in a cohort of pediatric TBI patients and how these variables are related to parameters of intracranial pathophysiology.

**Methods:**

A retrospective analysis was performed on pediatric severe TBI patients admitted to Addenbrookes Hospital Paediatric Intensive Care Unit (PICU) between January 2001 and December 2013. Demographic, outcome, systemic physiological, and cerebral autoregulatory data were extracted for patients who had received continuous invasive monitoring (ICM+, Cambridge Enterprise, Cambridge, UK). Data were analyzed using a mixed linear model.

**Results:**

Forty-four patients with an average age of 12.2 years were admitted to the PICU with a TBI requiring invasive neurosurgical monitoring. Thirty-two patients (73%) survived, with favorable outcomes in 62%. The mean (SD) intracranial pressure (ICP) was 17.6 + 9.0 mmHg, MAP was 89.7 + 9.0 mmHg, and pressure-reactivity index (PRx) was −0.01 + 0.23 a.u. The mean (SD) serum lactate was 2.2 (3.3) mmol/L. and the mean (SD) serum glucose was 6.1 (1.6) mmol/L. Early hyperglycemia was strongly associated with both PRx (Pearson correlation 0.351, *p* < 0.001) and ICP (Pearson correlation 0.240, *p* = 0.002) death (*p* = 0.021) and impaired cerebral autoregulation (*p* = 0.02). There was a strong association between ICP and serum lactate (*p* = 0.001).

**Conclusion:**

Increases in systemic glucose are associated with impaired cerebrovasular autoregulation after severe pediatric TBI. Moreover, deranged blood glucose is a marker of poor prognosis. Further studies are required to delineate putative mechanisms of hyperglycemia induced cerebral harm.

## Introduction

In the context of trauma, primary brain injury occurs due to cellular and extracellular matrix disruption from direct mechanical forces at the time of the traumatic incident. Primary brain injury then initiates a complex cascade of secondary molecular and vascular mechanisms culminating in inflammation, edema, impaired cerebral autoregulation, blood–brain barrier disruption, intracranial hypertension, reduced cerebral perfusion, and ultimately neuronal cell death. Secondary brain injury persists for weeks and may contribute to a further loss of potentially viable cerebral tissue, ultimately worsening neurological outcome (1). While primary brain injury is unpredictable and irreversible, the sequelae of secondary brain injury may be modified by prevention or minimization of recognized exacerbating systemic insults, such as hypotension, hypoxia, and hyperglycemia (2).

Hyperglycemia occurs frequently in the pediatric traumatic brain injury (TBI) population and the occurrence of elevated blood glucose values has been linked to increased mortality and worse neurological outcomes (3–6). While it is not entirely clear whether this association is due to a direct deleterious effect of hyperglycemia or simply a marker of illness severity, there is an increasing body evidence from the adult TBI population that ascribe a putative pathological role to raised blood glucose (2).

In a retrospective observational study of adult TBI our group has recently demonstrated an association between elevated blood glucose and impaired cerebral pressure-reactivity index (PRx) (7). Cerebral pressure reactivity is a fundamental component of cerebral autoregulation, whereby cerebrovascular resistance is altered in response to changes in cerebral perfusion pressure (CPP) (8). The PRx is a surrogate measure of cerebral pressure reactivity and calculated as the correlation between arterial blood pressure (MAP) and intracranial pressure (ICP) (9). A negative correlation implies active pressure reactivity while a positive correlation implies a “pressure passive,” impaired pressure reactivity. Previous studies have shown that a positive PRx is associated with disturbance of cerebral autoregulation (10, 11). Importantly, PRx has also been demonstrated to independently predict outcome after TBI in children (12, 13).

While epidemiological studies have demonstrated that the incidence of hospitalization and fatal brain injury is disproportionately high in children (14), somewhat surprisingly, the interplay between glycemia, markers of secondary neurological insult, and outcome remains poorly described in the pediatric population. The primary objective of this study was to determine associations between systemic glucose, cerebral pressure reactivity (PRx), and outcome in a cohort of pediatric TBI patients. Secondary objectives were to determine associations between systemic lactate, PRx and ICP.

## Materials and Methods

### Patients

This is a retrospective observational study of all pediatric patients admitted to an Intensive Care Unit in Addenbrooke’s Hospital, Cambridge with severe TBI from January 2001 to December 2015 inclusive. Consecutive patients with a clinical need for ICP monitoring were included for analysis. The insertion of an intracranial monitoring device is part of routine clinical practice and as such did not require ethical approval. The analysis of data within this study for the purposes of service evaluation was approved by the Cambridge University Hospital NHS Trust, Audit and Service Evaluation Department (Ref: 2143) and did not require ethical approval or patient consent.

Inclusion criteria were as follows: (1) TBI-related pathology, confirmed on CT or MRI, (2) severe injury (GCS < 8) failing to demonstrate significant early clinical improvement (i.e., poor neurology on sedation hold), and (3) requirement for invasive monitoring of ICP and mean arterial pressure (MAP). Patients were excluded if there was suspicion of non-accidental injury. Multi-modality monitoring was commenced at the earliest possible opportunity following arrival to the ICU and was terminated when sedation was lifted and the child either began to waken or died.

Pre-hospital data were recorded from the ambulance service records. Hypoxia was defined as saturations under 96% on arrival of the crew. Hypotension was defined as age-specific hypotension on arrival of the crew.

Patients were managed according to current TBI guidelines (15). Interventions were aimed at keeping ICP < 20 mmHg using a tiered treatment protocol of positioning, sedation, muscle paralysis, moderate hyperventilation, ventriculostomy, osmotic agents, and induced hypothermia. CPP was maintained >50–60 mmHg using intravenous fluids, vasopressors, and inotropes. Glucose management was achieved with a continuous intravenous insulin infusion targeting a blood glucose between 6 and 10 mmol/L. Clinical outcome was determined using the Glasgow Outcome Scale at 6 months (1—death, 2—persistent vegetative state, 3—severe disability, 4—moderate disability, 5—good recovery) (16). A favorable outcome was defined as a GOS ≥ 4.

### Data Acquisition and Analyses

Intracranial pressure was monitored with an intraparenchymal microsensor inserted into the right frontal cortex (Codman ICP MicroSensor, Codman and Shurtleff, Raynham, MA, USA) and arterial blood pressure (MAP) was monitored in the radial or femoral artery with a zero level at the right atrium (Baxter Healthcare CA, USA; Sidcup, UK). End-tidal carbon dioxide (CO_2_) data were collected from the ventilator.

Data were sampled at 100 Hz with proprietary data acquisition software (ICM+, Cambridge Enterprise, Cambridge, UK) and stored for subsequent analysis. Data were collected on each day of invasive monitoring until the fifth day post ictus. Cerebrovascular PRx was calculated as a moving Pearson correlation coefficient between 30 consecutive, 10-s averaged values of MAP and corresponding ICP signals (with 80% overlap of data). Averages over 10 s were used to suppress the influence of the pulse and respiratory frequency wave components.

Arterial blood samples were taken at 8 a.m. and 8 p.m. daily for the measurement of arterial glucose and lactate. All blood samples were analyzed by the Core Biochemical Assay Laboratory at Addenbrookes Hospital, Cambridge. Meso Scale Diagnostics (MD, USA) assays were standardized against an approved reference preparation (IFCC calibration). Six hours of time-averaged cerebrovascular data (CPP, ICP, PRx, and end-tidal CO_2_) were assessed for each measurement of arterial glucose and lactate.

### Statistical Analyses

Raw data were screened and cleared of artifacts and then examined for normality prior to analysis. The cohort was dichotomized into survivors and non-survivors. Ordinal data are presented as medians (IQR) and continuous data as means (SD). Differences in physiological values between survivors and non-survivors were interrogated with the Mann–Whitney *U*-test. The significance level was set to 0.05, and all tests were two-tailed and unadjusted for multiple comparisons. Bivariate correlation analyses (Pearson coefficient) were calculated between glucose, CPP, ICP, and PRx. Correlations are zero-order and unadjusted for multiple comparisons.

A repeated measures mixed effect model was generated to evaluate associations between systemic glucose and lactate, and intracerebral biochemical and pressure parameters with plasma glucose and lactate as covariates, time as a fixed effect and patient ID as a random effect. The model was adjusted for the following; age, injury characteristics (hypotensive and hypoxic episodes), ICP, PRx, and outcome. All data analyses were performed on SPSS version 21.0 software (SPSS Inc., Chicago, IL, USA). All statistical tests were performed with α ≤ 0.05 (two-tailed).

## Results

**Figure 1:**
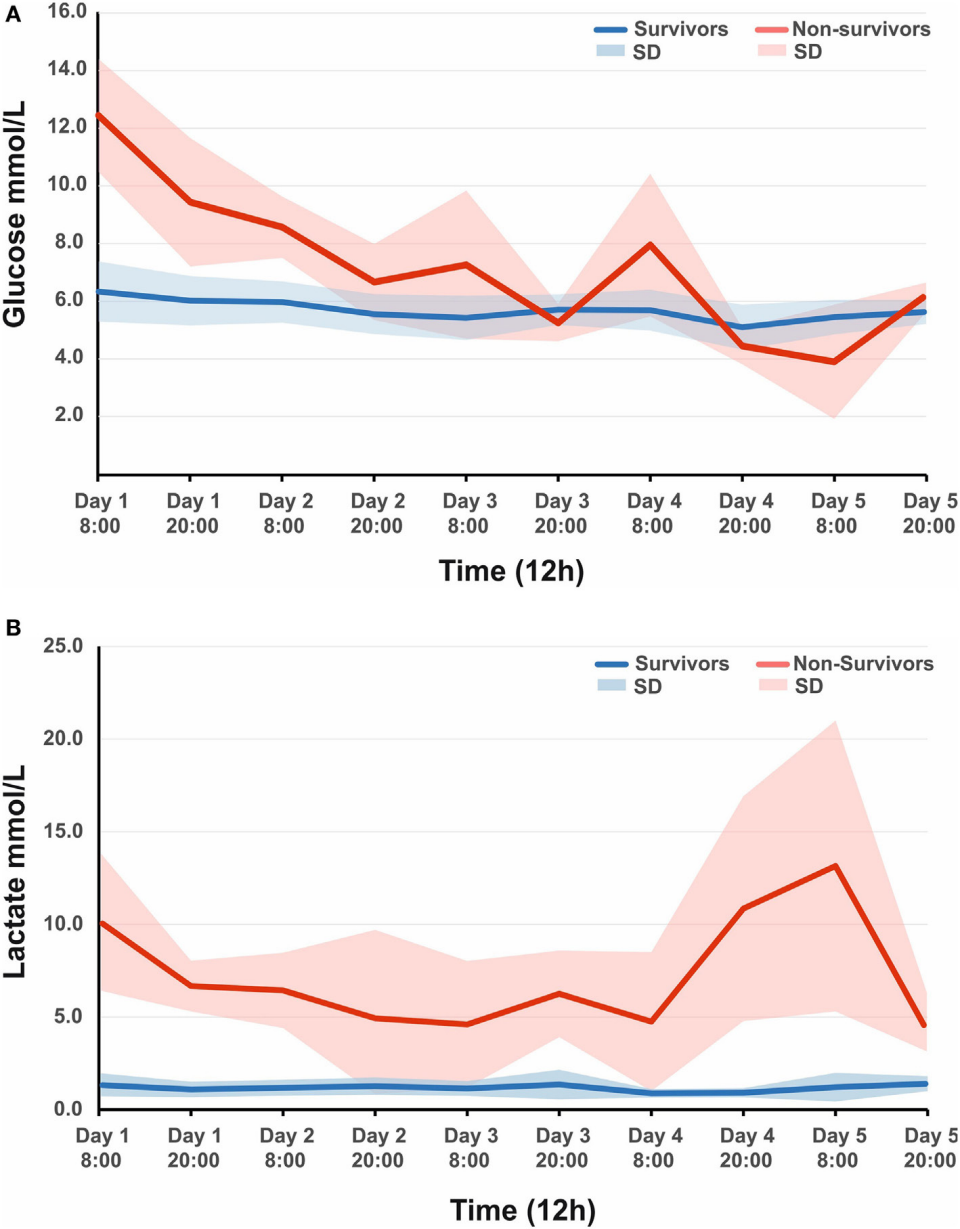
**(A)** Observed mean arterial glucose (SD) in pediatric traumatic brain injury (TBI) patients during the first 5 days since ictus, stratified by fatal outcome. **(B)** Observed mean arterial lactate (SD) in pediatric TBI patients during the first 5 days since ictus, stratified by fatal outcome.

A total of 44 patients with an average age of 12.2 years were admitted to the Paediatric Intensive Care Unit with a TBI requiring invasive neuro-monitoring (Table 1). Thirty-two patients (73%) were alive at 6 months, and 27 (62%) were deemed to have a favorable outcome. Thirty patients (68%) sustained an isolated head injury with the others having poly trauma. The incidence of poly trauma had no significant impact on outcome. All ICP wires were placed after sedation hold demonstrated poor neurology. This was usually within 6 h of injury. No patients were excluded on the basis of the timing of ICP insertion. Prior to the injury two children had mild learning disabilities, and one had attention deficit hyperactivity disorder. All patients were maintained at normothermia, none of them were cooled to hypothermia. Five patients had external ventricular drain inserted. Two patients had a decompressive craniectomy. 82% of patients had vassopressor/inotrope support. 64% of patients had insulin infusions in an attempt to control glucose levels. Mean physiologic monitoring values during the first 5 days since ictus are shown in Table 2. The mean (SD) ICP was 17.6 (9.0) mmHg, MAP was 89.7 (9.0) mmHg and PRx was −0.01 (0.22) a.u. The mean (SD) serum lactate was 2.2 (3.3) mmol/L. The mean (SD) serum glucose was 6.1 (1.6) mmol/L. The observed evolution of serum glucose and lactate over the first 5 days post-TBI has been visualized in Figures 1A,B.

**Table 1:** Demographics of pediatric patients presenting with a traumatic brain injury.

	Survived (*n* = 32)	Non-survivors (*n* = 12)	*p*-Value
Age, mean ± SD	12.1 + 5.1	12.5 + 5.7	0.45
Male (%)	26(62)	8(66)	0.62
Admission GCS, median (range)	9 (3–13)	3 (3–9)	0.03
Motor score	6 (1–6)	1 (1–5)	0.02
Pupils			
*Reactive (%)*	91	25	0.01
*Fixed Unilaterally (%)*	6	25	0.04
*Fixed Bilaterally (%)*	3	50	0.01
Hypoxia	6	33	0.45
Hypotension	6	17	0.15
Lactate, mean ± SD	1.9 ± 1.7	6.9 ± 3.1	0.21
Glucose, mean ± SD	6.4 ± 1.9	13.1 ± 3.9	0.02
ICP, mean ± SD	16.3 ± 4.1	24.8 ± 19.5	<0.001
PRx, mean ± SD	−0.03 ± 0.16	0.10 ± 0.43	0.01

*ICP, intracranial pressure; PRx, pressure- reactivity index*.

**Table 2:** Mean values (SD) and correlations of physiologic variables during intracranial monitoring during the first 5 days since ictus.

	Day 1–5 (*n* = 44)	95% CI	*p* Value
Arterial glucose in mmol/L (SD)	6.1 (1.6)		
Arterial lactate in mmol/L (SD)	2.2 (3.3)		
PRx a.u. (SD)	−0.01 (0.23)		
ICP mmHg (SD)	17.6 (9.0)		
MAP mmHg (SD)	89.7 (9.0)		
Glucose versus PRx (Pearson coefficient)	0.351*	0.2.04–0.497	<0.001
Lactate versus PRx (Pearson coefficient)	0.284*	0.133–0.434	<0.001
Glucose versus ICP (Pearson coefficient)	0.240*	0.088–0.392	0.002
Lactate versus ICP (Pearson coefficient)	0.294*	0.144–0.444	<0.001

*ICP, intracranial pressure; CPP, cerebral perfusion pressure; PRx, pressure reactivity; MAP, mean arterial pressure*.

*p value less than 0.05.

### Comparison of Demographic and Physiological Parameters between Survivors and Non-Survivors

Demographic and physiological data for survivors and non-survivors are presented in Table 1. The admission GCS in those who survived was 9 (3–13) at presentation compared to 3 (3–9) in non-survivors (*p* = 0.03). More significantly, the motor score was 6 (1–6) in survivors and 1 (1–5) in non-survivors (*p* = 0.02). The majority of survivors had bilateral reactive pupils (91%) versus 25% in non-survivors (*p* = 0.01). Only 6% of survivors had a unilaterally fixed pupil compared to 25% of non-survivors (*p* = 0.04), whereas 3% of survivors had bilaterally fixed pupils versus 50% of non-survivors (*p* = 0.01). There was no significant difference between the incidence of pre-hospital hypoxia or hypotension between survivors and non-survivors (7 versus 33%; *p* = −0.45 and 7 versus 17%; *p* = 0.15, respectively).

### Correlations between Systemic Glucose, Cerebral Autoregulation, and Outcome

**Figure 2:**
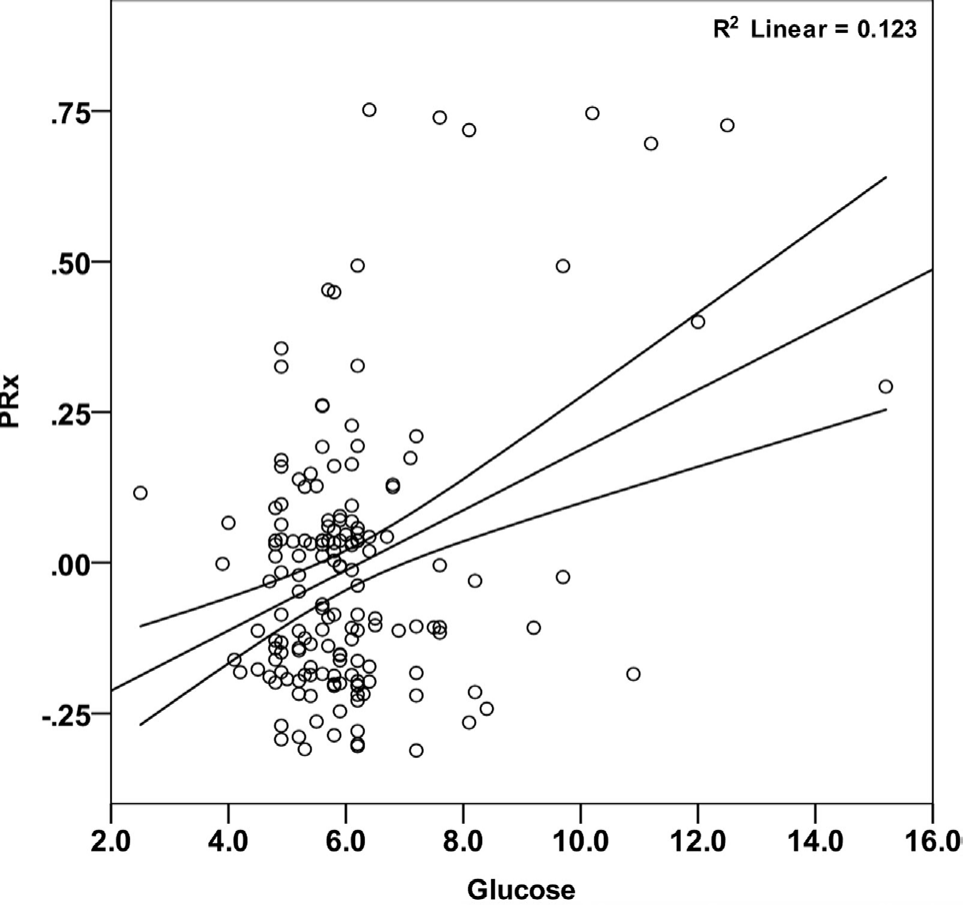
Correlation between mean arterial glucose and PRx in pediatric traumatic brain injury patients during the first 5 days since ictus. There was a significant positive relationship between blood glucose and PRx (Pearson correlation = 0.351; *p* < 0.001). Each data point represents the mean of the available arterial glucose concentration and PRx measurements during the first 5 days since ictus.

Mean arterial glucose concentration for each patient during the first 5 days from ictus was significantly correlated with mean PRx (Pearson correlation 0.351, *p* < 0.001; Table 2; Figure 2) and mean ICP (Pearson correlation 0.240, *p* = 0.002; Table 2). To account for the repeated measures for each patient in each outcome group, a linear mixed effects model analysis was used. The linear mixed effects model fitting identified a significant effect of PRx (*p* = 0.016) and non-survivors (*p* = 0.021) on arterial glucose. The interaction of these two factors (PRx versus Outcome) was non-significant (*p* = 0.124). Further modeling showed no additional contribution to model fit from age (*p* = 0.886), hypoxia and hypotension on admission (*p* = 0.408 and *p* = 0.488), or ICP (*p* = 0.593). Only daily mean arterial glucose concentration during the first 2 days post-TBI correlated significantly with mean PRx in the mixed effects model (*p* < 0.001), i.e., increases in systemic glucose correlated with a worse state of cerebral pressure reactivity.

### Correlations between Systemic Lactate, Cerebral Autoregulation, and Outcome

Mean arterial glucose concentration for each patient during the first 5 days from ictus was significantly correlated with mean PRx (Pearson correlation 0.284, *p* < 0.001; Table 2) and mean ICP (Pearson correlation 0.294, *p* < 0.001; Table 2). The linear mixed effects model fitting did not demonstrate correlations between serum lactate and PRx (*p* = 0.108) and hypoxia on admission (*p* = 0.87). However, a significant effect of ICP (*p* = 0.001) and hypotension on admission (*p* = 0.013) on arterial lactate was observed. The temporal evolution of lactate did not differ between fatal and non-fatal patients after adjustment in the mixed model (*p* = 0.318).

## Discussion

The key finding of this observational study is a significant positive relationship between arterial glucose concentration and cerebral pressure reactivity in pediatric TBI patients. Secondary observations are (i) a strong association between increasing blood glucose and mortality and (ii) a positive relationship between serum lactate and ICP.

Cerebral autoregulation is the physiologic mechanism that protects the brain against detrimental variations in cerebral blood flow (17), it is frequently impaired following severe TBI and is associated with worse functional outcomes following pediatric TBI (18, 19). Independently, poor glycemic control has been demonstrated to be linked to worse outcomes in pediatric TBI (20). Interestingly, the CHiP study (Control of hyperglycemia in pediatric intensive care) demonstrated no difference in outcome when comparing tight glycemic control versus conventional methods (21). Nevertheless, this study included multiple pathologies including trauma. By using the cerebral perfusion index as a surrogate measure of cerebral autoregulation, this is the first study to report an association between glycemia and impaired cerebral autoregulation in a pediatric TBI population. These findings are consistent with the association between glycemia and cerebral pressure reactivity in an adult TBI (7) and strengthen the external validity of these results as children are less likely to have any pre-existing systemic vascular pathology that could result in impaired cerebral autoregulation (22).

Traumatic brain injury initiates a dramatic systemic stress response with the release of cortisol, catecholamines, and glucagon leading to excessive hepatic gluconeogenesis and peripheral insulin resistance. The hyperglycemia attributed to these metabolic derangements is further exacerbated by therapeutic interventions, such as the administration of exogenous catecholamines and enteral or parenteral nutrition. Resultantly, hyperglycemia occurs frequently in patients with TBI, even in pediatric patients who were glucose tolerant prior to the traumatic insult (3–6). Consistent with these previous studies, we report an association between elevated blood glucose concentration and mortality. Despite these findings, there remains a lack of evidence guiding the management of hyperglycemia in the pediatric TBI population which is reflected in a marked disparity in insulin regimens and glucose targets worldwide (23). Furthermore, it remains unclear whether hyperglycemia is truly deleterious or is simply a marker of illness severity; however, it is generally accepted the extremes of hyperglycemia (>10 mmol/L) should be avoided (24).

Manifold putative mechanisms whereby glucose causes neurological harm have been described including induction of oxidative stress pathways (25, 26), generation of pro-inflammatory transcription factors (27), and disruption of blood–brain barrier integrity (28) predisposing to oedma and cell death (29, 30). It has also been suggested that hyperglycemia may impair vascular function; exogenous glucose reduces regional cerebral blood flow (31, 32) which may be linked to impaired endothelial function and smooth muscle vasoconstriction (33, 34). While we acknowledge that association does not prove causation, our findings support the concept that hyperglycemia post-TBI may contribute to impaired cerebral autoregulation.

Finally, we report an association between ICP and serum lactate. While this may just be a marker of severity of illness, the concept that lactate, or rather acidosis, may alter ICP merits consideration. The presence of high levels of lactate within the cerebrovascular circulation can result in localized vasodilation, extravasation, and worsening edema with a potential increase in ICP (35, 36). Furthermore, the ubiquitous disruption of blood–brain barrier integrity following TBI allows the inappropriate passage of lactate into the brain parenchyma where it can exert an osmotic load and contribute to brain edema (37). Future mechanistic studies employing tracer labeled lactate and cerebral microdialysis are required to help clarify this relationship further.

### Limitations

The current study has several important limitations. Primarily, it is possible that the observed relationships could be due to the effect of a confounding physiologic variable. Additionally, serum glucose levels were collected only twice daily. Since severe TBI in pediatric patients is relatively rare, strong multi-center collaborative groups are required to evaluate mechanisms underlying the relationships observed with the ultimate aim of guiding clinical practice to improve patient outcomes.

## Conclusion

Consistent with data from the adult TBI population, we have demonstrated that elevations in blood glucose may impair cerebrovascular reactivity in pediatric TBI, supporting the requirement for adequate glucose control in the first few days post insult. Further studies are warranted to delineate mechanisms linking glycemia and impaired cerebral autoregulation.

## Ethics Statement

The analysis of data within this study for the purposes of service evaluation was approved by the Cambridge University Hospital NHS Trust, Audit and Service Evaluation Department (Ref: 2143) and did not require ethical approval or patient consent.

## Author Contributions

AY designed the study, performed the analysis, and wrote the paper, HA, JD, and MGuilfoyle performed the analysis, HF provided patient care and directed the methods, MGarnett provided patient care and directed the methods, MC provided supervision of the analysis, PS provided supervision of the analysis, MP provided expertise on glucose control, SA designed the project and provided supervision of the analysis, and PH designed the study and oversaw the project.

## Conflict of Interest Statement

ICM+ software is licensed by Cambridge Enterprise Ltd.; PS and MC have financial interest in a part of the licensing fee. All other authors declare that the research was conducted in the absence of any commercial or financial relationships that could be construed as a potential conflict of interest. The reviewer FC declared a shared affiliation, though no other collaboration, with the authors to the handling editor, who ensured that the process nevertheless met the standards of a fair and objective review.
