# Corrigendum: Glycemia Is Related to Impaired Cerebrovascular Autoregulation after Severe Pediatric Traumatic Brain Injury: A Retrospective Observational Study

**DOI:** 10.3389/fped.2017.00245

**Published:** 2017-11-24

**Authors:** Adam M. H. Young, Hadie Adams, Joseph Donnelly, Mathew R. Guilfoyle, Helen Fernandes, Mathew R. Garnett, Marek Czosnyka, Peter Smielewski, Mark Plummer, Shruti Agrawal, Peter J. Hutchinson

**Affiliations:** ^1^Division of Academic Neurosurgery, Department of Clinical Neurosciences, Addenbrooke’s Hospital, University of Cambridge, Cambridge, United Kingdom; ^2^Neurosciences Critical Care Unit, Addenbrooke’s Hospital, University of Cambridge, Cambridge, United Kingdom; ^3^Department of Paediatric Intensive Care, Addenbrooke’s Hospital, University of Cambridge, Cambridge, United Kingdom

**Keywords:** brain, injury, acute, glucose, lactate

## Missing Funding

In the original article, we neglected to include the funder(s) for this article. We gratefully acknowledge financial support as follows. Research support: the Medical Research Council (MRC, Grant Nos. G0600986 ID79068 and G1002277 ID98489) and the National Institute for Health Research Biomedical Research Centre (NIHR BRC) Cambridge (Neuroscience Theme; Brain Injury and Repair Theme). Authors’ support: PH—NIHR Research Professorship, Academy of Medical Sciences/Health Foundation Senior Surgical Scientist Fellowship and NIHR Cambridge BRC. AY is supported by an NIHR Academic Clinical Fellowship. JD is supported by a Woolf Fisher Scholarship. The authors apologize for this error and state that this does not change the scientific conclusions of the article in any way.

The original article was updated.

## Conflict of Interest Statement

The authors declare that the research was conducted in the absence of any commercial or financial relationships that could be construed as a potential conflict of interest.

